# Genomic analysis of community-associated multidrug-resistant *Klebsiella quasipneumoniae* subsp. *similipneumoniae* and the identification of the ST2059-KL1 clone in the U.S.

**DOI:** 10.1128/spectrum.01107-26

**Published:** 2026-07-10

**Authors:** Jianping Jiang, Austin J. Terlecky, Elena Shashkina, Meghan W. Starolis, Liang Chen, Barry N. Kreiswirth

**Affiliations:** 1 Center for Discovery and Innovation, Hackensack Meridian Health 3139, Nutley, New Jersey, USA; 2 Quest Diagnostics 7172 , Secaucus, New Jersey, USA; 3 School of Pharmacy and Pharmaceutical Sciences, University at Buffalo, Buffalo, New York, USA; Zhejiang University, Hangzhou, China

**Keywords:** ST2059, KL1, multidrug-resistant, *Klebsiella quasipneumoniae* subsp. similipneumoniae

## Abstract

*Klebsiella quasipneumoniae* subsp. *similipneumoniae* is an important member of the *K. pneumoniae* species complex (KpSC) and is increasingly reported as multidrug-resistant (MDR) in healthcare- and community-associated infections. Since clinical laboratories do not routinely distinguish *K. quasipneumoniae* subsp. *similipneumoniae* from *K. pneumoniae*, national prevalence estimates, particularly for MDR, are lacking. In this study, a total of 2,006 community-associated MDR KpSC isolates were collected from 42 U.S. states, with 30 *K. quasipneumoniae* subsp. *similipneumoniae* isolates originating from 12 states identified using whole genome sequencing. All isolates were resistant to ceftriaxone and exhibited high rates of resistance to other antimicrobial agents, including ampicillin-sulbactam (56.7%, 17/30), levofloxacin (75.9%, 22/29), and trimethoprim-sulfamethoxazole (53.3%, 16/30). Notably, five isolates were also carbapenem-resistant. Genomic analysis resolved 10 sequence types (STs), with ST2059 (*n* = 13) and ST414 (*n* = 9) predominating. Ceftriaxone resistance in most isolates (90%, 27/30) was conferred by an extended-spectrum β-lactamase gene, predominantly *bla*_CTX-M-15_ (73.3%, 22/30); the remaining isolates carried either a carbapenemase (*bla*_KPC-3_) or an AmpC β-lactamase (*bla*_CMY-2_). Nanopore sequencing identified *bla*_CTX-M-15_ harbored on two types of IncFIB(Kpn3) antimicrobial resistance (AMR) plasmids, either with or without the conjugative *tra* gene cluster. Interestingly, the KL1 locus, associated with canonical hypervirulent *K. pneumoniae* strains, was detected in all ST2059 isolates. Further analysis of public genomic data showed that the KL1 locus is widely distributed across KpSC. KL1 phylogenetic analyses indicated frequent intrasubspecies recombination but limited intersubspecies exchange of KL1. The identification of the dominant MDR *K. quasipneumoniae* subsp. *similipneumoniae* KL1-ST2059 clone in the U.S. underscores the importance of ongoing genomic surveillance.

**IMPORTANCE:**

*Klebsiella quasipneumoniae* subsp. *similipneumoniae* is an underrecognized member of the *Klebsiella pneumoniae* species complex that is frequently misidentified in clinical laboratories, leading to an incomplete understanding of its role in antimicrobial resistance. In this study, we used large-scale genomic surveillance of community-associated multidrug-resistant isolates across the U.S. to identify this subspecies as a reservoir of clinically relevant resistance plasmids. Notably, we detected a widely distributed ST2059 lineage carrying the K1 capsular locus, a feature traditionally associated with hypervirulent *K. pneumoniae*. These findings highlight the convergence of resistance and virulence-associated traits in an overlooked species and underscore the need for genomic surveillance to monitor emerging high-risk lineages in community settings.

## INTRODUCTION

Multidrug-resistant (MDR) *Klebsiella pneumoniae*, especially strains that produce extended-spectrum β-lactamases (ESBLs) or carbapenemases, cause severe healthcare-associated infections, such as pneumonia and bacteremia, and represent a major public health concern (1). ESBL-producing and carbapenem-resistant *K. pneumoniae* represent major global public health threats because of the limited availability of effective antimicrobial agents (1).

Within the genus *Klebsiella*, several species share approximately 95% average nucleotide identity with *K. pneumoniae sensu stricto*, forming a monophyletic clade known as the *K. pneumoniae* species complex (KpSC) (2). The KpSC comprises five closely related species: *K. pneumoniae sensu stricto*, *Klebsiella quasipneumoniae, Klebsiella variicola*, *Klebsiella quasivariicola*, and *Klebsiella africana* (3). More recently, isolates genotyped as *K. quasipneumoniae* were further classified into two subspecies: *K. quasipneumoniae* subsp. *quasipneumoniae* and *K. quasipneumoniae* subsp. *similipneumoniae* (2). Both subspecies are increasingly recognized as causes of human infection (4). Of clinical concern are reports of *K. quasipneumoniae* subsp. *similipneumoniae* identified as the etiologic agent of neonatal septicemia in China (5) and as the cause of a neonatal bacteremia outbreak at a tertiary hospital in Nigeria (6). Notably, the true prevalence of this subspecies is likely underestimated because routine clinical laboratories do not differentiate members of the KpSC (7).

Capsular serotype K1, encoded by the KL1 capsule locus, is a virulence-associated feature and commonly linked to sequence type (ST) 23 hosts (8). ST23 K1 hypervirulent strains are common in Asian countries and cause pyogenic infections, with recent reports highlighting the emergence of antimicrobial resistance (9, 10). The K1 capsule is not restricted to *K. pneumoniae sensu stricto* and has been identified in other KpSC species, including *K. quasipneumoniae* subsp. *similipneumoniae*. For example, in 2017, a hypervirulent *K. quasipneumoniae* subsp. *similipneumoniae* isolate was reported from a patient with chronic liver disease in India belonging to ST2320 and carrying the K1 capsule (11). In 2022, a hypervirulent, carbapenem-resistant K1 *K. quasipneumoniae* subsp. *similipneumoniae* strain harboring a virulence plasmid was reported in China (12).

In this study, we collected 2,006 ceftriaxone non-susceptible *K. pneumoniae* isolates and identified 30 *K. quasipneumoniae* subsp. *similipneumoniae* strains from 12 states across the U.S. We characterized their microbiologic and genomic features and found that MDR in *K. quasipneumoniae* subsp. *similipneumoniae* was encoded on IncFIB(Kpn3) plasmids carrying multiple resistance determinants. Interestingly, we also identified a dominant clone ST2059 with the KL1 locus. Phylogenetic analysis of the KL1 locus across the KpSC revealed extensive inter- and intra-subspecies recombination. These findings provide insight into the spread of *K. quasipneumoniae* subsp. *similipneumoniae* in the U.S. and highlight the need for genomic surveillance of this species.

## MATERIALS AND METHODS

### The study

Between July 2023 and July 2024, 10 Quest Diagnostics regional laboratories identified over 10,000 ceftriaxone-non-susceptible (Minimum inhibitory concentration [MIC] ≥2 µg/mL) *K. pneumoniae* isolates. From these, 2,006 isolates representing diverse clinical specimen types, including urine, blood, respiratory, and other sources, were selected for whole genome sequencing. All selected isolates were MDR according to definitions in the literature (13). Bacterial identification and susceptibility testing were performed by each contributing microbiology laboratory using Vitek MS and/or Vitek 2 ID and AST cards (N808/XN15) (bioMérieux, Durham, NC, USA) with the exception of one laboratory (Chantilly, VA). Identification at that site was performed with Biotyper (Bruker, Billerica, MA, USA) for identification, followed by susceptibility using Vitek 2 ID and AST cards (N808/XN15) (bioMérieux, Durham, NC, USA). “Community-associated” refers to isolates collected through Quest Diagnostics from predominantly outpatient and community-based settings (>80% from non-hospitalized patients). This designation was based on the sampling framework rather than strict clinical criteria due to incomplete epidemiological data.

### Whole genome sequencing and analysis

Whole genome sequencing was performed as previously described using the Illumina NextSeq 500 platform with 2 × 150 bp paired-end reads (14). Illumina raw sequencing reads were trimmed using Trimmomatic v0.39 (15). Draft genomes were assembled using SPAdes v3.13.0 (16). The multilocus sequence type (MLST) was determined *in silico* using MLST (https://github.com/tseemann/mlst). Additionally, seven strains were further sequenced using the Oxford Nanopore MinION platform and assembled via Unicycler 0.4.9 (17) using both short and long sequencing reads. Kleborate v2.3 (18) was employed to identify members of the KpSC and to screen genome assemblies for capsular type identification and detection of virulence loci, including hypermucoidy genes *rmpADC*, *rmpA2,* and siderophore systems, such as yersiniabactin (*ybt*), colibactin (*clb*), aerobactin (*iuc*), salmochelin (*iro*), as well as their associated mobile elements (*ICEKp*). Resistance genes were determined by AMRFinderPlus v3.10.20 (19) and ARIBA v2.14.6 (20). Capsular polysaccharide synthesis locus (KL) and lipopolysaccharide O antigen synthesis locus (OL) types were typed by Kapative v2.0.9 (21). Plasmid replicons were identified by ABRicate v1.01 (https://github.com/tseemann/abricate) by using the PlasmidFinder (22) database with 90% identity and 80% query coverage cutoffs. The circular alignment of plasmids with complete sequences was generated by using BRIG v0.95 (23). The linear alignment of KL1 regions was visualized by using Easyfig v2.1 (24).

### Phylogenetic analysis

Core-genome alignments were used to construct the phylogenetic tree. The genes were predicted by using Prokka v1.14.5 (25). The core-genome alignments based on the concatenation of core genes were obtained using panaroo v1.5.0 (26) using default parameters and core genes defined as those being present in more than 98% of the genomes. For the capsular region phylogeny, the KL1 capsular locus sequence of NTUH-K2044 (accession no. AB924547), which corresponds to the CPS region only, was used as the reference. Raw reads or contigs were mapped to the reference KL1 locus region by using Snippy v4.6.0 (https://github.com/tseemann/snippy). The SNP density plot was generated by using ggplot2 as previously described (27), with the SNPs called within KL1 region. Maximum-likelihood phylogenetic trees were constructed by using FastTree v2.1.11 (model GTR + GAMMA) (28). The tree was visualized by using iTOL v6 (29).

### Quantification of mucoviscosity

Six ST2059 *K. quasipneumoniae* subsp. *similipneumoniae* isolates were randomly selected for mucoviscosity testing. The mucoviscosity was measured as previously described (30). Briefly, overnight cultures were diluted 1:10 in fresh medium and incubated for 6 h to reach exponential growth. The cultures were adjusted to an OD_600_ of 1.0, followed by centrifugation at 1,000 × *g* for 5 min. The OD_600_ of the supernatant was subsequently measured to determine the hypermucoid index. Each assay was performed in triplicate.

## RESULTS

### Antimicrobial susceptibility profile of 30 MDR *K. quasipneumoniae* subsp. *similipneumoniae*

Between July 2023 and July 2024, 10 Quest Diagnostics regional laboratories collected 2,006 unique ceftriaxone-nonsusceptible (MIC ≥2 µg/mL) *K. pneumoniae* isolates (one per patient) from 42 U.S. states for whole-genome characterization. Of these, 1,928 were *K. pneumoniae sensu stricto*, 45 were *K. quasipneumoniae,* 32 were *K. variicola,* and one was *K. quasivariicola*. Among *K. quasipneumoniae*, 30 were further identified as *K. quasipneumoniae* subsp. *similipneumoniae*. The 30 isolates originated from 13 states (Fig. S1), and most were recovered from urine samples among outpatients with urinary tract infections (UTIs; 90%, 27/30). All isolates were MDR, with 100% resistance to cefazolin (30/30) and ceftriaxone (30/30) (Table 1). Resistance rates to cefepime and ceftazidime were 43.3% (13/30) and 39.3% (11/28), respectively. Notably, five isolates were carbapenem-resistant. High rates of nonsusceptibility were also observed for ampicillin-sulbactam (56.7%, 17/30), ciprofloxacin (60.0%, 18/30), amoxicillin-clavulanate (34.5%, 10/29), levofloxacin (75.9%, 22/29), and trimethoprim-sulfamethoxazole (53.3%, 16/30) (Table 1), underscoring the limited effectiveness of commonly used agents for UTIs caused by MDR *K. quasipneumoniae* subsp. *similipneumoniae*.

**TABLE 1:** Antimicrobial susceptibility profiles of all isolates against 13 antimicrobial agents^*a*^

ID	CFZ	CRO	FEP	CAZ	IMP	MEM	AMC	SAM	TZP	CIP	LEV	GEN	SXT
BK69047	R	R	R	R	R	R	R	R	R	R	R	R	R
BK73804	R	R	S	R	R	R	ND	R	R	I	I	I	S
BK69129	R	R	S	S	S	S	I	R	S	R	I	R	R
BK70116	R	R	S	R	S	S	I	R	S	R	I	S	S
BK72166	R	R	R	I	S	S	R	R	S	R	R	R	R
BK69846	R	R	S	S	S	S	S	S	S	I	I	S	S
BK69936	R	R	S	S	S	S	S	S	S	S	I	S	R
BK70037	R	R	S	S	S	S	S	S	S	S	I	S	R
BK70390	R	R	S	S	S	S	S	S	S	S	I	S	R
BK70521	R	R	S	S	S	S	S	S	S	I	I	S	R
BK70560	R	R	S	I	S	S	S	I	S	R	R	S	S
BK72349	R	R	R	ND	S	S	S	R	R	R	R	S	R
BK72659	R	R	R	R	S	S	S	S	I	I	I	S	R
BK73279	R	R	S	S	S	S	S	S	ND	S	I	S	R
BK73305	R	R	S	I	I	S	S	I	S	S	ND	S	R
BK73450	R	R	R	R	S	S	R	R	I	R	R	R	R
BK73481	R	R	R	R	S	S	S	R	I	R	R	S	R
BK73532	R	R	R	R	S	S	S	S	S	S	S	S	R
BK73900	R	R	R	R	R	R	R	R	R	S	S	S	S
BK69016	R	R	S	S	S	S	I	S	S	S	S	S	S
BK70087	R	R	S	S	S	S	S	S	S	R	R	S	S
BK70515	R	R	S	S	S	S	S	S	S	R	I	S	S
BK70585	R	R	S	ND	S	S	S	S	S	S	S	S	S
BK70909	R	R	S	I	S	S	S	I	S	R	R	S	R
BK72682	R	R	R	R	S	S	S	R	S	R	R	S	S
BK73171	R	R	R	R	R	R	R	R	R	S	S	S	R
BK73508	R	R	R	S	S	S	S	S	S	R	I	S	S
BK73816	R	R	R	I	S	S	S	R	S	S	S	S	S
BK70437	R	R	S	S	R	R	R	R	S	S	S	S	S
BK70399	R	R	R	R	S	S	I	R	I	I	I	S	S

^*a*^ CFZ, cefazolin; CRO, ceftriaxone; FEP, cefepime; CAZ, ceftazidime; IMP, imipenem; MEM, meropenem; AMC, amoxicillin-clavulanate; SAM, ampicillin-sulbactam; TZP, piperacillin-tazobactam; CIP, ciprofloxacin; LEV, levofloxacin; GEN, gentamicin; SXT, trimethoprim-sulfamethoxazole; R, resistant; I, intermediate; S, susceptible; ND, not determined.

### Genomic characteristics and phylogeny

**Fig 1:**
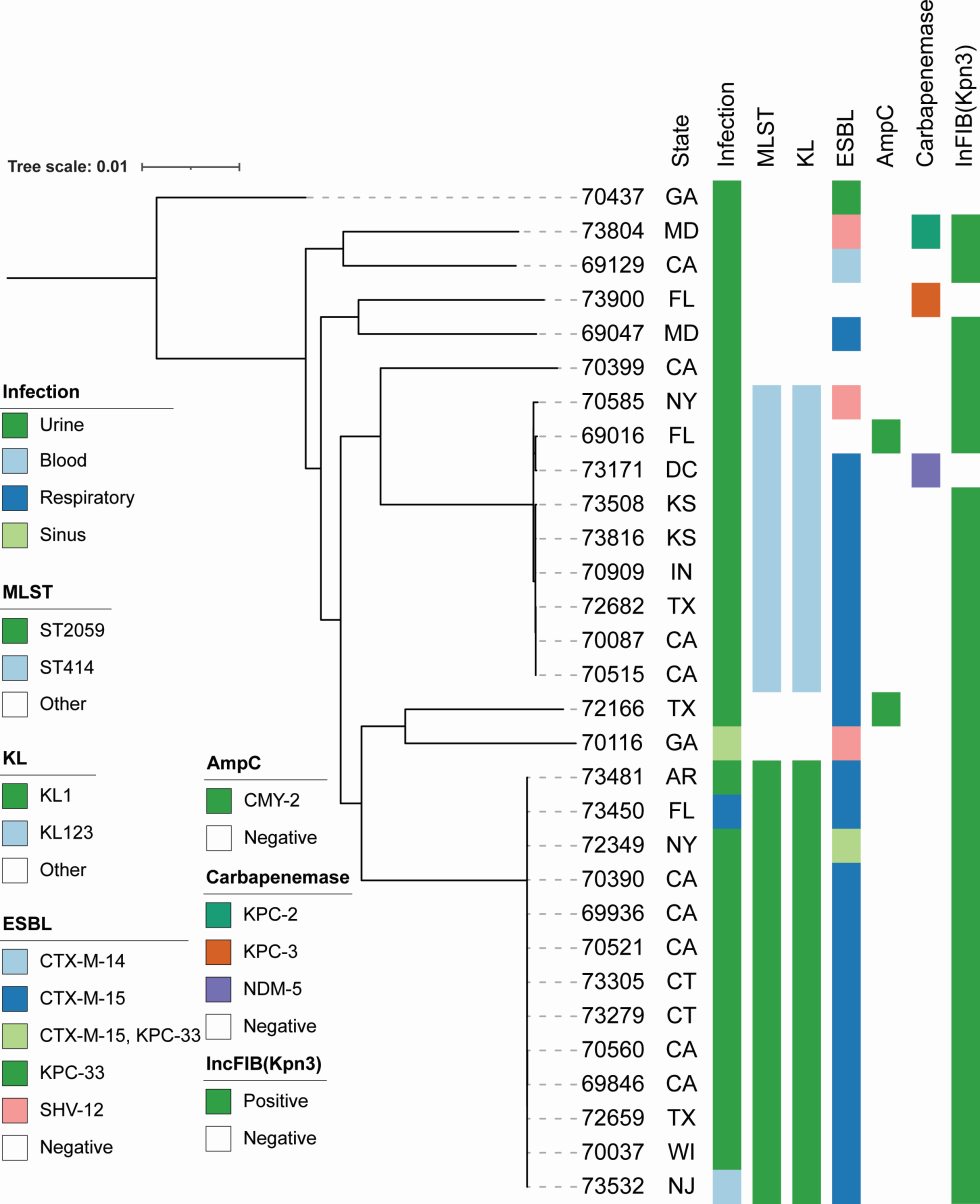
Phylogenetic analysis of 30 MDR-*K. quasipneumoniae* subsp. *similipneumoniae* isolates.

Genomic analysis identified 10 STs, with ST2059 (*n* = 13) and ST414 (*n* = 9) being the most prevalent, accounting for 73.3% (22/30) of all the *K. quasipneumoniae* subsp. *similipneumoniae* isolates (Fig. S1). Phylogenetic analysis showed that isolates from different STs were distantly related (Fig. 1). Within each of the two dominant STs, isolates were recovered from multiple states, inconsistent with localized outbreaks. Notably, all ST2059 isolates harbored the KL1 capsular locus, showing 97% coverage and 94% identity to the KL1 locus of NTUH-K2044 (based on ST2059 isolates with complete genome sequences), which is associated with hypervirulent ST23 *K. pneumoniae*.

Analysis of AMR determinants revealed that most isolates (90%, 27/30) carried an ESBL gene, with *bla*_CTX-M-15_ predominating (73.3%, 22/30) (Fig. 1; Table S1), and three isolates additionally carried a carbapenemase or an AmpC β-lactamase. The remaining isolates encoded either a carbapenemase (3.3%, 1/30) or an AmpC β-lactamase (3.3%, 1/30). The mechanism of ceftriaxone resistance in one isolate was unknown. Genes conferring resistance to fluoroquinolones (*qnr*, 80%, 24/30), trimethoprim (*dfrA*, 53.3%, 16/30), and sulfonamides (*sul*, 80%, 24/30) were common, consistent with the high rates of nonsusceptibility to these agents. Tetracycline (*tetA*/*tetD*, 33.3%, 10/30) and macrolide (*mphA*, 36.7%, 11/30) resistance genes were also identified, further reducing oral treatment options. Among the five carbapenem-resistant isolates, three carried carbapenemase genes (*bla*_KPC-2_, *bla*_KPC-3_, or *bla*_NDM-5_); one was carbapenem resistance as a result of expressing an ESBL in a background that lacked a functional OmpK36 porin protein; and one had an undetermined mechanism. Neither *mgrB* alterations, which confer colistin resistance, nor known resistance-associated QRDR mutations were identified among the 30 isolates, suggesting that fluoroquinolone resistance may be mediated by plasmid-borne genes *qnr* and additional mechanisms. In most isolates (90%, 27/30), an IncFIB(Kpn3) replicon was identified (Fig. 1).

### AMR plasmids

To further investigate AMR plasmids in MDR *K. quasipneumoniae* subsp. *similipneumoniae*, we selected seven isolates (ST2059 [*n* = 3], ST414 [*n* = 2], ST1834 [*n* = 1], and ST1699 [*n* = 1]) for additional whole genome analysis using the Oxford Nanopore long-read sequencing platform. The hybrid Illumina/Nanopore sequencing data were analyzed to completely assemble the genomes and plasmids of each strain (Table 2). Each harbored 1–4 plasmids ranging from 4 to 275 Kb. IncFIB(Kpn3) plasmids carrying most of the AMR determinants were identified in all seven isolates, with sizes ranging from 81 to 275 kb. In addition, IncFII(pHN7A8) and ColRNAI plasmids were detected in three isolates, and three isolates carried one or two untypeable plasmids, underscoring the remarkable plasmid diversity and genetic plasticity of these isolates.

**TABLE 2:** Genomic characteristics of seven completely sequenced isolates

	BK72659	BK70560	BK69936	BK69016	BK73816	BK72166	BK69129
MLST	ST2059	ST2059	ST2059	ST414	ST414	ST1834	ST1699
State	TX	CA	CA	FL	KS	TX	CA
Date	2024 Jan	2023 Oct	2023 Aug	2023 Jul	2024 Jun	2023 Dec	2023 Aug
Site of infection	Urine	Urine	Urine	Urine	Urine	Urine	Urine
Genome size	5.3Mb	5.6Mb	5.4Mb	5.6Mb	5.3Mb	5.4Mb	5.3Mb
#Plasmids	1	3	2	4	1	4	2
Plasmid replicon [size]	IncFIB(Kpn3) [81 Kb]	IncFIB(Kpn3) [196 Kb], IncFIB(pQil) [126 Kb], Unknown [5Kb]	IncFIB(Kpn3) [209 Kb], Unknown [4 Kb]	IncFIB(Kpn3) [212 Kb] , IncFII(pHN7A8) [71 Kb], Unkown [52 Kb], Unkown [34 Kb]	IncFIB(Kpn3) [163 Kb]	IncFIB(Kpn3) [170 Kb], IncFII(pHN7A8) [71 Kb], ColRNAI [4 Kb], ColRNAI [4 Kb]	IncFIB(Kpn3)/ IncFII(pHN7A8)/ColRNAI [275 Kb], ColRNAI [4 Kb]
KL	KL1	KL1	KL1	KL123	KL123	KL159	KL52
OL	O5	O5	O5	O3/O3a	O3/O3a	OL101	OL103
AMR genes	*bla*CTX-M-15*, mphA, sul1, aadA2, dfrA12, sul2, strA, strB, qnrS1, catA*	*tetA, bla*CTX-M-15*, sul2, strA, strB, qnrS1*	*tetA, bla*CTX-M-15*, mphA, sul1, aadA2, dfrA12, sul2, strA, strB, qnrS1, catA2*	*tetD, bla*CMY-2	*sul2, strA, strB, bla*CTX-M-15 (chromosome)*, bla*CTX-M-15 (plasmid)	*sul2, strA, strB, bla*TEM-1B*, bla*CTX-M-15*, dfrA14, bla*_OXA-1'_ *aac(6*'*)Ib-cr, tetA, qnrB1, aac (3)-IIa, bla*_CMY-2_	*bla*CTX-M-14*, aac (3)-IId, qnrS1, sul1, dfrA1, tetA, tetD, catA2, bla*TEM-1B*, strB, strA, sul2*

**Fig 2:**
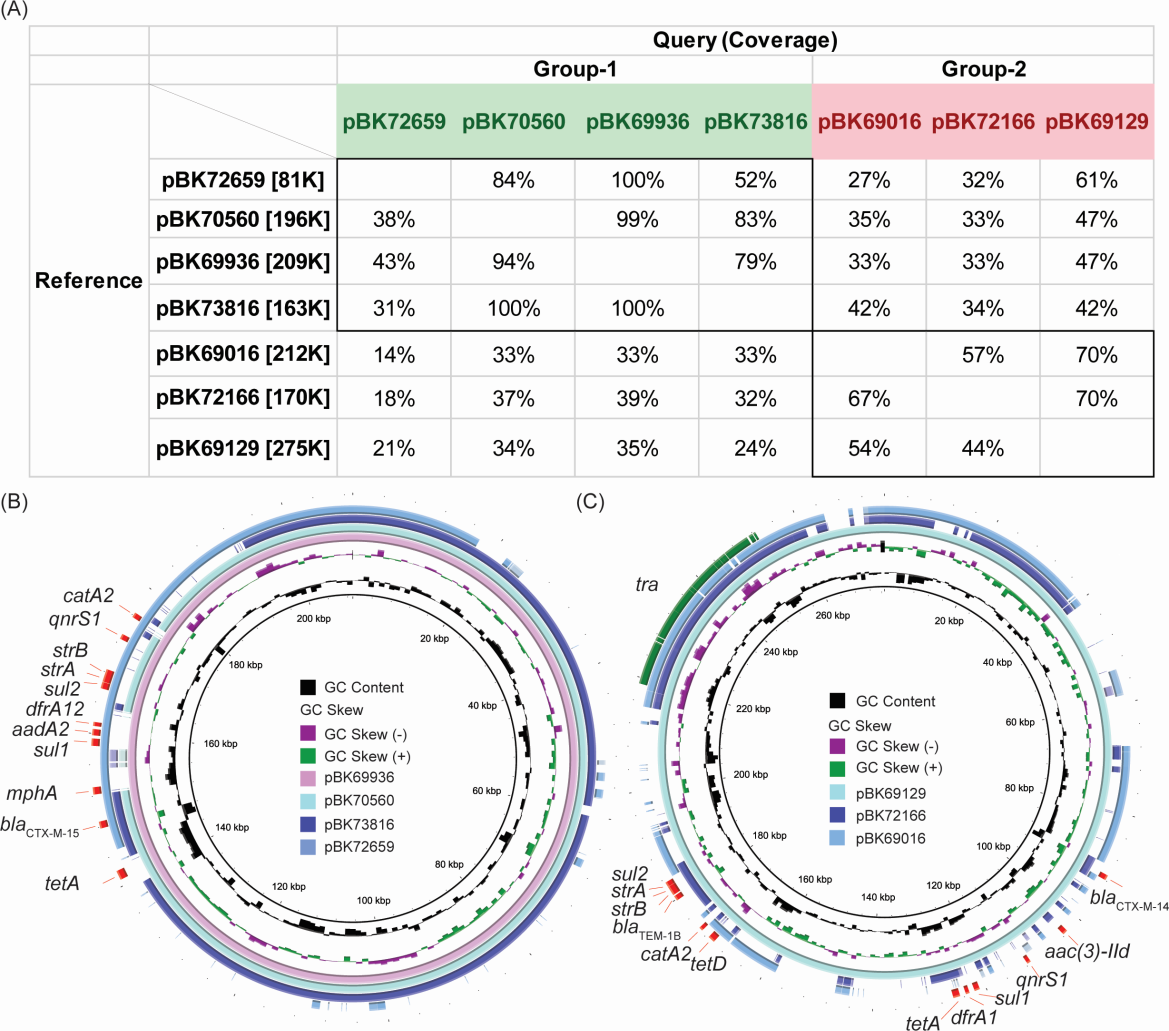
MDR plasmids in MDR-*K. quasipneumoniae* subsp. *similipneumoniae* isolates. (**A**) Matrix of pairwise comparison of MDR plasmids. (**B**) pKPN plasmid without *tra* locus. (**C**) pKPN plasmid with *tra* locus. MDR genes were indicated in red. Conjugative-related genes (*tra*) were indicated in green.

Because most AMR genes resided on the IncFIB(Kpn3) plasmids, we analyzed these in detail. Each isolate harbored *bla*_CTX-M-15_ on an IncFIB(Kpn3) plasmid except for pBK69126, which carried *bla*_CTX-M-14_. Comparative analyses resolved two plasmid groups (Group 2) with the mean intra-group alignment covering 75% and 60%, respectively, and inter-group coverage of 29%–39% (Fig. 2A). The groups were distinguished principally by the presence of the conjugative *tra* locus in Group 2 plasmids, suggesting potential transferability based on genomic prediction (Fig. 2B and C). AMR determinants were clustered within discrete resistance regions. Despite broadly similar backbones within each group, the plasmids exhibited structural variation, particularly across AMR islands, even among isolates of the same ST. For example, among Group 1 plasmids, *qnrS1* was present in pBK69936, pBK70560, and pBK72659 but absent from pBK73816 (Fig. 2B); likewise, among Group 2 plasmids, *tetA* was present in pBK69129 and pBK72166 but absent from pBK69016 (Fig. 2C).

Comparing the IncFIB(Kpn3) plasmids of *K. quasipneumoniae* subsp. *similipneumoniae* with plasmids available in the NCBI revealed that both Group 1 and Group 2 plasmids share high sequence similarity with the reported plasmids (GenBank accession numbers CP085867.1 [31] and KY271404.1 [32]) from *K. pneumoniae sensu stricto*, indicating extensive intra-subspecies plasmid transfer.

### Evolutionary origin of capsular locus KL1 in ST2059 *K. quasipneumoniae* subsp. *similipneumoniae*

Since all 13 ST2059 isolates in our cohort harbored the KL1 capsular locus, a well-characterized capsular type associated with invasion and immune evasion of ST23 *K. pneumoniae*, we next investigated the evolutionary history of KL1 to determine its origin in *K. quasipneumoniae* subsp. *similipneumoniae*. A survey of NCBI genomes (accessed in July 2025, Table S2) identified 2,472 KL1-positive *K. pneumoniae* genomes spanning 60 known STs. These comprise *K. pneumoniae sensu stricto* (*n* = 2,391), *K. quasipneumoniae* subsp. *similipneumoniae* (*n* = 58), *K. quasipneumoniae* subsp. *quasipneumoniae* (*n* = 18), and *K. variicola* subsp. *variicola* (*n* = 5). Among KL1-positive genomes, ST23 was dominant (86.9%, 2,149/2,472), followed by ST700 (2.1%, 52/2,472) and ST82 (1.4%, 34/2,472), all typed to *K. pneumoniae sensu stricto*. Alignment of the KL1 locus from representative strains showed high conservation across these subspecies (Fig. S2).

**Fig 3:**
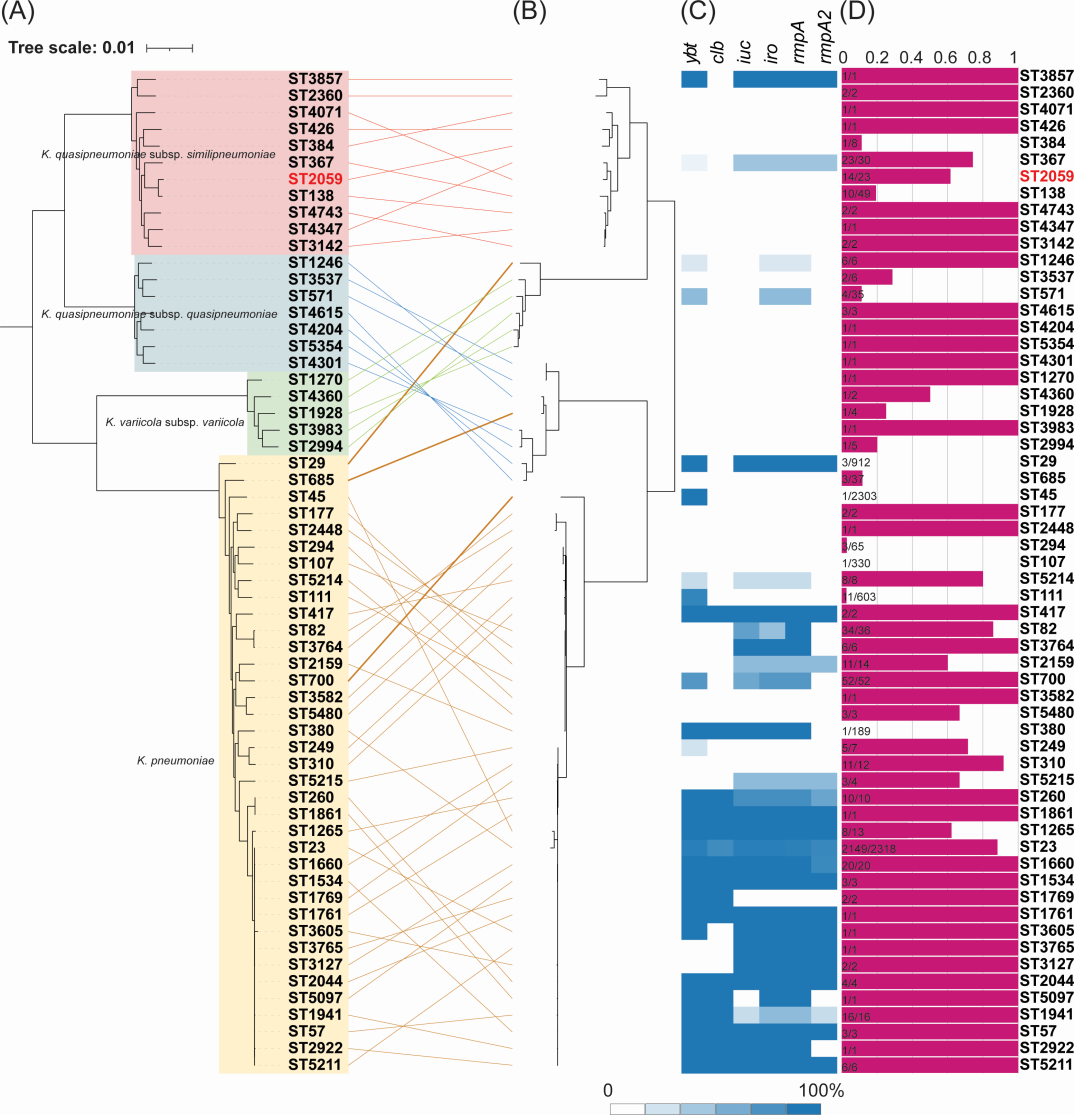
Evolution of the KL1 capsule locus in KpSC. (**A**) The core genome phylogenetic tree of KL1 clones in KpSC. *K. quasipneumoniae* subsp. *similipneumoniae* was indicated in red; *K. quasipneumoniae* subsp. *quasipneumoniae* in blue; *K. variicola* subsp. *variicola* in green; and *K. pneumoniae* in yellow. The inter-subspecies exchange of KL1 was highlighted in bold. (**B**) Phylogenetic tree of the KL1 capsule locus in KpSC. (**C**) The heatmap of virulence genes in each clone. (**D**) The ratio of KL1 in each clone.

Core-genome phylogeny of all KL1-bearing isolates indicated that *K. variicola* subsp. *variicola* was most closely related to *K. pneumoniae sensu stricto*, whereas *K. quasipneumoniae* subsp. *similipneumoniae* and *K. quasipneumoniae* subsp. *quasipneumoniae* were closely related to each other (Fig. 3A), which is consistent with a previous study (33). Phylogenetic analysis of the KL1 locus region clustered most strains by subspecies (Fig. 3B), suggesting that KL1 evolved with speciation and that cross-subspecies exchange of KL1 is rare. Nonetheless, inter-subspecies exchange of KL1 was identified (Fig. 3B). For example, ST29 *K. pneumoniae sensu stricto* shared KL1 with *K. quasipneumoniae* subsp. *quasipneumoniae*, and ST685 *K. pneumoniae sensu stricto* shared KL1 with *K. variicola* subsp. *variicola*—a finding consistent with the SNP density plots shown in Fig. S3. We also detected a ~3 Kb recombinant segment in ST700 *K. pneumoniae sensu stricto*, likely derived from *K. quasipneumoniae* subsp. *similipneumoniae* (Fig. S3), which rendered the ST700 clone an outlier on the KL1 tree (Fig. 3B). Phylogenetic analysis of KL1 showed greater similarity between *K. quasipneumoniae* subsp. *quasipneumoniae* and *K. pneumoniae sensu stricto*, supporting independent acquisition of KL1 by the two *K. quasipneumoniae* subspecies. The KL1 in ST2059 was likely acquired through recombination within the subspecies, as evidenced by the local clustering of *K. quasipneumoniae* subsp. *similipneumoniae* on KL1 tree.

Most virulence determinants, particularly those typically found on virulence plasmids, were observed in *K. pneumoniae sensu stricto*, including the prototypically hypervirulent ST23 and closely related STs such as ST1660 and ST1265 (Fig. 3C; Table S3). However, virulence genes were also present in KL1-positive *K. quasipneumoniae* (e.g., ST571 and ST367), although no virulence genes have been detected in the ST2059 strains. Analysis of capsule distribution within STs containing >10 genomes showed that KL1 was the predominant capsule (present in >50% of genomes) in more than half of these STs (10/19), including ST23 (92.7%, 2,149/2,318) in *K. pneumoniae sensu stricto* and ST367 (76.7%, 23/30) in *K. quasipneumoniae* subsp. *similipneumoniae* (Fig. 3D; Table S3). KL1 was present in 14 of 23 ST2059 genomes, indicating that ST2059 has become a reservoir of KL1. The presence of KL1 in ESBL-encoding ST2059 strains highlights the importance of monitoring the co-occurrence of MDR and virulence-associated features.

### Mucoviscosity of KL1-ST2059 *K. quasipneumoniae* subsp. *similipneumoniae* isolates

**Fig 4:**
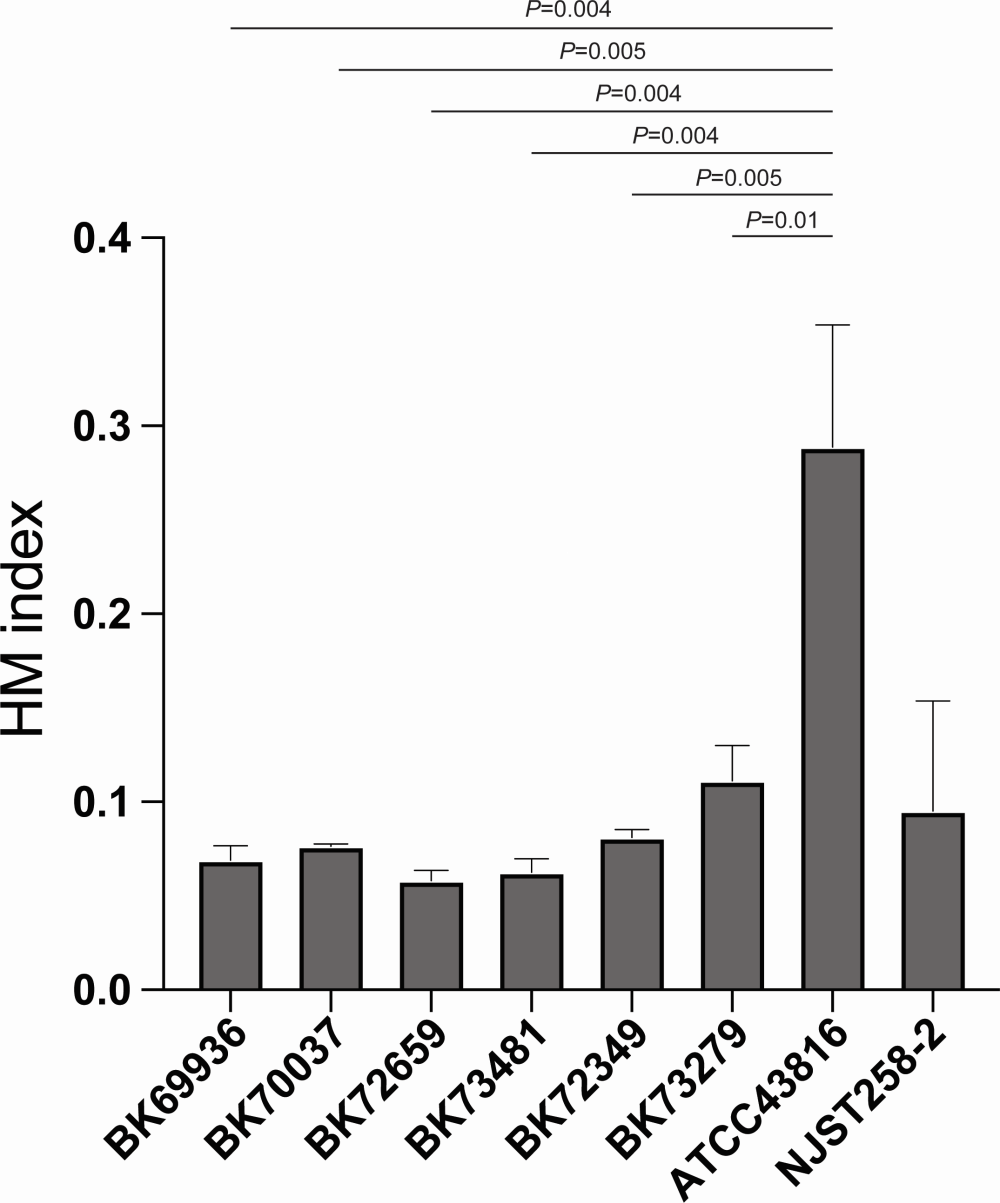
The hypermucoid index of the six KL1-ST2059 *K. quasipneumoniae* subsp. *similipneumoniae* isolates. The hypermucoid index of the six isolates was compared to those of ATCC 43816 and NJST258-2 using *t*-tests, respectively.

We next assessed the mucoviscosity of six KL1-ST2059 *K. quasipneumoniae* subsp. *similipneumoniae* isolates and compared the results with those for *K. pneumoniae* ATCC43816 (hypermucoviscous) and *K. pneumoniae* NJST258-2 (low mucoviscosity) reference strains. All KL1-ST2059 *K. quasipneumoniae* subsp. *similipneumoniae* isolates exhibited low mucoviscosity. The hypermucoid index of these isolates was significantly lower than that of the hypermucoviscous reference strain ATCC43816 and did not differ significantly from that of the low-mucoviscosity reference strain NJST258-2 (Fig. 4). The low mucoviscosity observed in KL1-ST2059 *K. quasipneumoniae* subsp. *similipneumoniae* isolates is likely attributable to the absence of mucoid regulators, such as RmpADC and RmpA2.

## DISCUSSION

In this study, we collected and sequenced 30 MDR *K. quasipneumoniae* subsp. *similipneumoniae* isolates originated from 12 states in the U.S. Two predominant STs, ST2059 (*n* = 13) and ST414 (*n* = 9), were identified in this collection. IncFIB(Kpn3) plasmids carrying multiple AMR genes, including *bla*_CTX-M-15_, were prevalent across these isolates. Notably, the virulence-associated capsular locus KL1 was present in all ST2059 isolates. Comparative genomic and phylogenetic analyses indicated that KL1 has disseminated to multiple members of the KpSC via recombination. These findings advance the epidemiologic characteristics of MDR *K. quasipneumoniae* subsp. *similipneumoniae* in the U.S. and highlight the importance of ongoing monitoring of MDR-KL1 convergence.

*K. quasipneumoniae* subsp. *similipneumoniae* is a recently recognized member of the KpSC, first formally described in 2014 (34). Now, it is increasingly recognized but still frequently misidentified as *K. pneumoniae sensu stricto* by conventional clinical microbiological methods, leading to underestimation of its true burden worldwide (7). The *K. quasipneumoniae* subsp. *similipneumoniae* clinical isolates have been recovered from multiple infections, including bloodstream, respiratory, urinary, and intra-abdominal infections, across diverse geographic regions (4, 12, 35), although its prevalence generally represents a minor proportion of KpSC (e.g., ~3% in a single-center survey in Singapore) (4). Because *K. quasipneumoniae* subsp. *similipneumoniae* remains underrecognized in most U.S. clinical laboratories, national prevalence estimates are lacking. Our data showed that *K. quasipneumoniae* subsp. *similipneumoniae* accounted for approximately 1.5% of isolates within our ceftriaxone-non-susceptible MDR KpSC collection, indicating a relatively minor contribution compared to *K. pneumoniae sensu stricto* in this high-risk subset. Although the numbers are small, it should be highlighted that the vast majority of the characterized *K. quasipneumoniae* subsp. *similipneumoniae* harbor IncFIB(Kpn3), the multidrug-resistant plasmid commonly found in the MDR *K. pneumoniae sensu stricto*.

In addition, and consistent with plasmid movement, a longitudinal genomic investigation demonstrated that *K. quasipneumoniae* subsp. *similipneumoniae* can persist in healthcare wastewater systems for years, undergo repeated AMR plasmid acquisitions, and enable it to reseed patient populations (36). Therefore, although *K. quasipneumoniae* subsp. *similipneumoniae* has not yet emerged as a dominant lineage, its ecological niche in healthcare settings and capacity to acquire and transfer AMR plasmids underscore its importance for infection control and surveillance.

*K. quasipneumoniae* subsp. *similipneumoniae* can readily acquire and harbor AMR determinants on mobilizable plasmids serving as a reservoir for multidrug resistance within the KpSC. Clinical isolates have been reported to carry ESBL genes such as *bla*_CTX-M_, *bla*_SHV_, and *bla*_TEM_, as well as carbapenemase genes, including *bla*_KPC_, *bla*_NDM_, and *bla*_OXA-48-like_ (36, 37). These genes are most often located on conjugative plasmids of the IncF, IncN, IncR, and IncX3 groups, which are widely disseminated among Enterobacterales (38, 39). Our study identified IncFIB(Kpn3) plasmids that harbored *bla*_CTX-M-15_ in most of the MDR *K. quasipneumoniae* subsp. *similipneumoniae*. The IncFIB(Kpn3) plasmids carried additional resistance determinants, including fluoroquinolone resistance genes and aminoglycoside-modifying enzymes within the AMR island, adding to their arsenal of multidrug resistance. The IncFIB(Kpn3) plasmids from *K. quasipneumoniae* subsp. *similipneumoniae* were also found in other KpSC species, including *K. pneumoniae sensu stricto* and *K. variicola*, indicating that AMR-associated IncFIB(Kpn3) plasmids have circulated among multiple KpSC subspecies. These findings underscore *K. quasipneumoniae* subsp. *similipneumoniae* as a competent host for AMR plasmids and an underestimated contributor to plasmid-mediated resistance spread in healthcare settings.

Capsular locus KL1 is a virulence-associated feature and is well-characterized in ST23 *K. pneumoniae sensu stricto*, a prototypically hypervirulent clone associated with community-onset invasive disease (40). Although ST23-KL1 predominates, we and others showed that KL1 is not restricted to ST23 and can be found in other STs and subspecies across the KpSC, presumably through capsular switching (41). In this study, we identified KL1 in additional lineages (e.g., ST700 and ST82) and in other KpSC taxa, including *K. quasipneumoniae* subsp. *similipneumoniae* and *K. quasipneumoniae* subsp. *quasipneumoniae*. We also found evidence that non-*K*. *pneumoniae sensu stricto* donors can serve as sources of KL1 through recombination; for example, ST29 *K. pneumoniae* appears to have acquired KL1 from *K. quasipneumoniae* subsp. *quasipneumoniae*, yielding ST29-KL1 strains. Together, these findings indicate that KL1 is a transferable capsule locus with broad host compatibility across the KpSC, enabling recurrent cross-taxon acquisition and supporting the occurrence of KL1-associated capsular convergence in MDR isolates beyond the classic ST23 lineage.

Capsule locus recombination and capsule switching are common across the KpSC (41). Interclonal recombination of the KL1 capsular locus may lead to phenotypic diversification and enhanced virulence expression in distinct *K. pneumoniae* lineages. For example, Huang et al. demonstrated that the KL1 capsule type alone in ST23 strains dictates immune evasion and *in vivo* virulence (42). Therefore, KL1 may also contribute to virulence in other clones, including ST2059 strains, although their virulence phenotypes will require further confirmation.

Given that most isolates were recovered from outpatient urinary tract infections and exhibited high resistance rates to commonly used oral agents such as trimethoprim-sulfamethoxazole and fluoroquinolones, empirical treatment options may become increasingly limited. Although some agents, including aminoglycosides and carbapenems, retained relatively preserved activity, these drugs are often less suitable for routine outpatient therapy because of toxicity concerns or the need for intravenous administration. These findings highlight the importance of ongoing antimicrobial susceptibility surveillance and support the need for susceptibility-guided therapy, particularly in regions with a high prevalence of multidrug-resistant KpSC isolates (43). In addition, the increasing dissemination of multidrug-resistant community-associated KpSC lineages may further complicate the clinical management of outpatient urinary tract infections and underscore the importance of antimicrobial stewardship.

This study has several limitations. First, the collection was restricted to ceftriaxone-non-susceptible MDR KpSC isolates, and therefore, the findings reflect this selected subset rather than the overall prevalence of *K. quasipneumoniae* subsp. *similipneumoniae*. Second, the sample size of *K. quasipneumoniae* subsp. *similipneumoniae* was limited and predominantly derived from outpatient urinary specimens, which may limit the generalizability of our findings to isolates from other infection sites or healthcare settings. However, the predominance of urine-derived outpatient isolates may also reflect the characteristics of community-associated *K. quasipneumoniae* subsp. *similipneumoniae* infections. Third, although Kaptive has been extended for use across the KpSC, it was primarily developed using *K. pneumoniae sensu stricto* genomes, and its performance in other subspecies of KpSC has been less extensively validated, which should be considered when interpreting capsular locus assignments. Fourth, the potential transferability of the plasmids was inferred based on the presence of conjugation-associated genes and plasmid sequence characteristics, but no conjugation experiments were performed for functional validation. In addition, the designation of “community-associated” was based on the fact that more than 80% of the isolates were recovered from patients treated outside the hospital setting. Finally, the potential contribution of the KL1 capsule locus to virulence in ST2059 *K. quasipneumoniae* subsp. *similipneumoniae* was not functionally validated. Future studies incorporating *in vivo* virulence models and functional assays are needed to clarify the biological significance of KL1 in the ST2059 background.

In conclusion, *K. quasipneumoniae* subsp. *similipneumoniae* is an emerging MDR pathogen in both community- and healthcare-associated infections. The identification of dominant *K. quasipneumoniae* subsp. *similipneumoniae* clones in the U.S., particularly those carrying the virulence-associated capsular locus KL1, underscores the importance of ongoing genomic surveillance.

## Data Availability

The genome sequences have been deposited in the DDBJ/ENA/GenBank under the bioproject PRJNA549322.

## SUPPLEMENTAL MATERIAL

Figure S1 spectrum.01107-26-s0001.tif Distribution of STs across different states.

Figure S2 spectrum.01107-26-s0002.tif Linear alignment of KL1 capsule locus region.

Figure S3 spectrum.01107-26-s0003.tif Recombination of capsular locus KL1 in KpSC.

Supplemental legends spectrum.01107-26-s0004.docx Legends for Figures S1 to S3.

Tables S1 to S3 spectrum.01107-26-s0005.xlsx Genomic metadata for isolates contained in this study.

ASM does not own the copyrights to Supplemental Material that may be linked to, or accessed through, an article. The authors have granted ASM a non-exclusive, world-wide license to publish the Supplemental Material files. Please contact the corresponding author directly for reuse.
