# Supplementary material for: Genomic analysis of community-associated multidrug-resistant Klebsiella quasipneumoniae subsp. similipneumoniae and the identification of the ST2059-KL1 clone in the U.S
Source: Microbiol Spectr. 2026 Jul 10;14(8):e01107-26. doi: 10.1128/spectrum.01107-26 (PMC13436224; doi:10.1128/spectrum.01107-26)
Supplement: Supplementary file 1 [file spectrum.01107-26-s0004.docx]

**Supplemental Figure Legend**

**Figure S1. Distribution of STs across different states.**

**Figure S2. Linear alignment of KL1 capsule locus region.** BK72659 is genotyped to ST2059 *K. quasipneumoniae* subsp. *similipneumoniae*; NTUH-K2044 (AP006725.1) is genotyped to ST23 *K. pneumoniae sensu stricto*; KP2011790 (CP066173.1) is genotyped to ST367 *K. quasipneumoniae* subsp. *similipneumoniae*; 22KM2707 (CP128466.1) is genotyped to ST1270 *K. variicola* subsp. *variicola*; and 2606_C1 (GCF_029437735.1) is genotyped to ST1246 *K. quasipneumoniae* subsp. *quasipneumoniae*.

**Figure S3. Recombination of capsular locus KL1 in KpSC.** The red box indicated the regional recombination.
